# Association between 8q24 rs6983267 polymorphism and cancer susceptibility: a meta-analysis involving 170,737 subjects

**DOI:** 10.18632/oncotarget.18960

**Published:** 2017-07-04

**Authors:** Man Zhu, Xue Wen, Xuefang Liu, Yingchao Wang, Chunzi Liang, Jiancheng Tu

**Affiliations:** ^1^ Department of Clinical Laboratory Medicine and Center for Gene Diagnosis, Zhongnan Hospital of Wuhan University, Wuhan, Hubei, China

**Keywords:** cancer, 8q24, rs6983267, polymorphism, meta-analysis

## Abstract

Published data on the association between 8q24 rs6983267 polymorphism and cancer risk are inconsistent. Thus, we conducted a meta-analysis to evaluate the relationship between rs6983267 polymorphism and cancer risk. We searched on PubMed, EMBASE, Web of Science and China National Knowledge Infrastructure (CNKI) up to November 1, 2016 for relevant studies. Odds ratios (ORs) and 95% confidence intervals (CIs) were used to estimate the strength of this association. We included 78 case-control studies with a total of 73,996 cases and 96,741 controls in this meta-analysis. The pooled results showed that rs6983267 polymorphism was significantly associated with increased risk of overall cancer in all genetic models (dominant model: OR = 1.19, 95% CI = 1.13–1.26; recessive model: OR = 1.19, 95% CI = 1.14–1.25; homozygous model: OR= 1.31, 95% CI = 1.23–1.40; heterozygous model: OR = 1.14, 95% CI = 1.10–1.19; allelic model: OR = 1.14, 95% CI = 1.11–1.18). Stratified analyses indicated that rs6983267 significantly increased the risk of colorectal cancer in Caucasians, prostate cancer in Caucasians and Asians, thyroid cancer in Caucasians and lung cancer in Asians. When studies were stratified by study quality, source of controls and genotyping method, significant associations were especially found in the high quality studies, the publication-based studies, the hospital-based studies, and the PCR-RFLP studies. Additional well-designed studies with large samples should be performed to validate our results.

## INTRODUCTION

Cancer has become a major public health problem. According to the GLOBOCAN 2012, approximately 14.1 million new cancer cases and 8.2 million cancer deaths were reported worldwide [1]. Epidemiological and biological evidence demonstrate that carcinogenesis is a complex process involving multiple environmental and genetic factors, although the etiology of carcinogenesis has not been fully elucidated.

Chromosomal 8q24 has emerged recently as a risk locus for various types of cancer among different ethnicities (Caucasians, Asians, and Africans) [2]. 8q24 has been described as a “gene desert”, since the 600-kbp gene-poor region appears to have little transcriptional activity. Despite this, accumulating evidence suggested that 8q24 may play an active role in carcinogenesis. For example, POU5F1P1, which was originally considered as a pseudogene, has been identified on 8q24. It is now supposed that POU5F1P1 can encode a functional protein which contributes to carcinogenesis by acting as a weak transcriptional activator [3]; MYC, which acts as a transcriptional activator involving cell growth, differentiation, apoptosis, and other intracellular responses, is significantly associated with 8q24 [4, 5]. It has been reported that the 8q24 region contains multiple enhancer elements which can activate transcription of the nearby oncogene MYC; 8q24 also contains several other genes that functionally participate in cancer development, including ectonucleotide pyrophosphatase/phosphodiesterase 2 gene (ENPP2) and nephroblastoma overexpressed gene (NOV). ENPP2 encodes a phospholipase, which stimulates tumor cell proliferation [6]. NOV encodes regulatory protein CCN3, which involves in cancer development [6]. Furthermore, MetaCore regulatory network analysis found that both NOV and ENPP2 were indirectly linked by MYC [7].

Genome-wide association studies (GWASs) have identified the polymorphism rs6983267 as a new susceptibility locus for several cancer types [8–11]. Polymorphism rs6983267 which locates on 8q24 is a G/T single nucleotide polymorphism (SNP). There are three genotypes of rs6983267: homozygous risk alleles (GG), homozygous non-risk alleles (TT), and heterozygous alleles (G/T). Previous studies by *Tuupanen et al*. [12] and *Sur et al*. [13] demonstrated that the risk allele (G allele) serves as a binding element for the enhancer protein TCF4/LEF1 which can accelerate the transcription of MYC *in vivo*. Since then, a great number of studies have been performed on this polymorphism with the risk of many cancers in different populations but have generated equivocal results. Up to now, a number of meta-analyses have been published and implied a possible association between rs6983267 polymorphism and cancer risk. Unfortunately, some meta-analyses have presented contradictory results. For instance, *Troutman et al*. [14] indicated that rs6983267 was significantly associated with a high risk for prostate cancer among Caucasians, Asians and Africans. However, *Zhu et al*. [15] demonstrated that rs6983267 was associated to prostate cancer among Caucasians and Asians. Intriguingly, in 2016, *Yang et al*. [16] observed this association among Caucasians only. Besides, such contradictions also existed in some other meta-analyses [17–19]. Of note, lack of further evaluation in different stratified analyses prevented comprehensive understanding in some recent meta-analyses [20, 21]. To better understand the precise relationship, we performed a comprehensive meta-analysis with increased statistical power.

## RESULTS

### Characteristics of eligible studies

**Figure 1:**
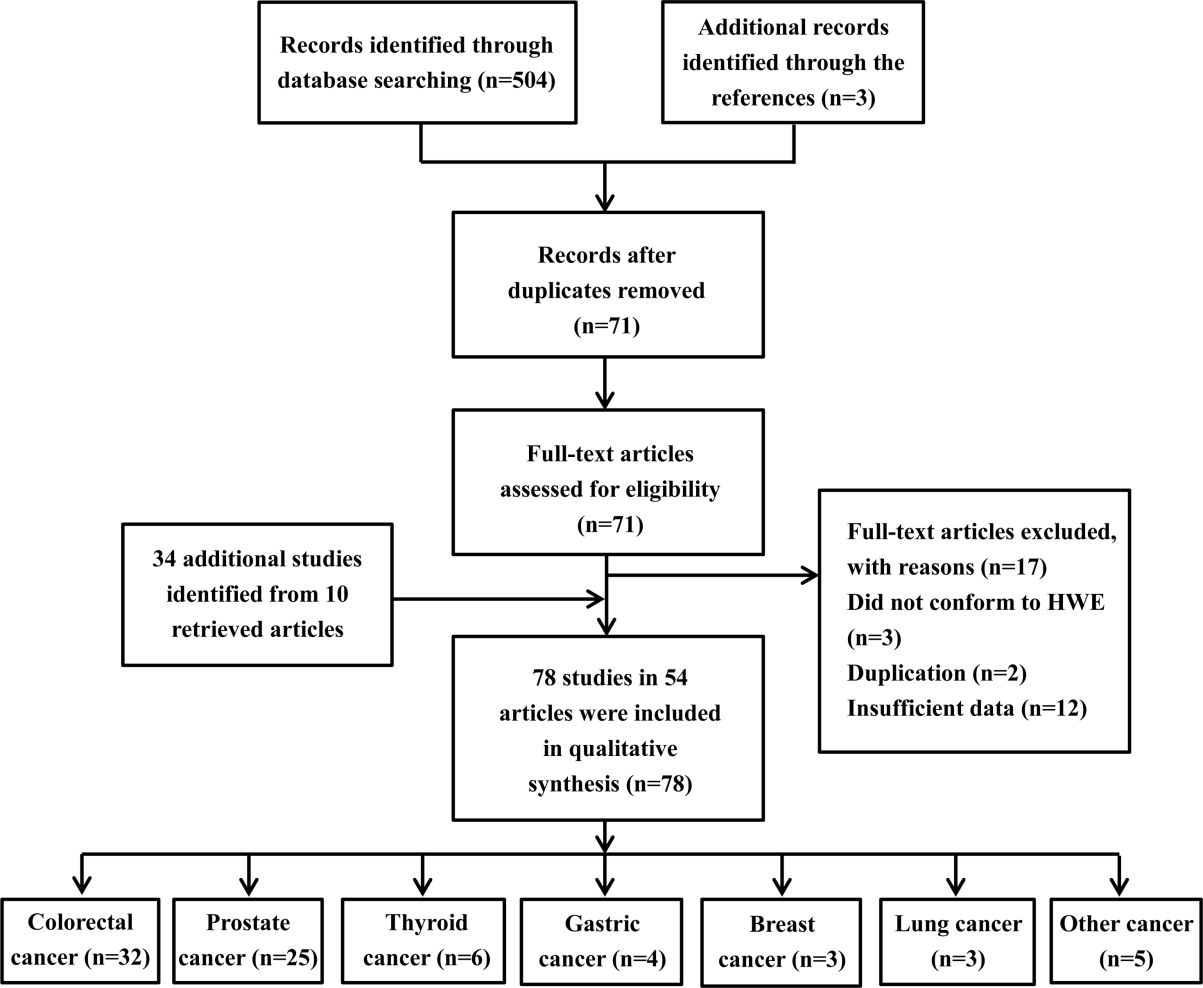
Flow chart of the process for study identification and selection

A total of 504 articles were preliminarily identified at first based on our selection strategy. We also identified three papers through the references. After scanning all of the abstracts, there were 71 articles that conformed to inclusion criteria. We excluded 12 articles that did not have completely extractable data [22–33], 3 articles were excluded because they did not conform to HWE [34–36], 2 studies were excluded because they were duplicated with previous publications [37, 38]. Thus, we included 54 independent records [8, 10, 12, 36, 39–89]. One study [52] was treated as 9 independent case groups because nine cancer types were studied. Moreover, we retrieved 25 separated investigations from 9 articles [8, 10, 39, 42, 54, 61, 74, 83, 88]. Finally, a total of 78 separate studies involving 73,996 cases and 96,741 controls were included in our meta-analysis. Among them, there were 32 studies on colorectal cancer, 25 on prostate cancer, 6 on thyroid cancer, 4 on gastric cancer, 3 on breast cancer, 3 on lung cancer and 5 on other cancers. Figure 1 describes the process for the study. The baseline characteristics of all eligible studies are summarized in Supplementary Table 1.

### Meta-analysis of the overall population

**Figure 2:**
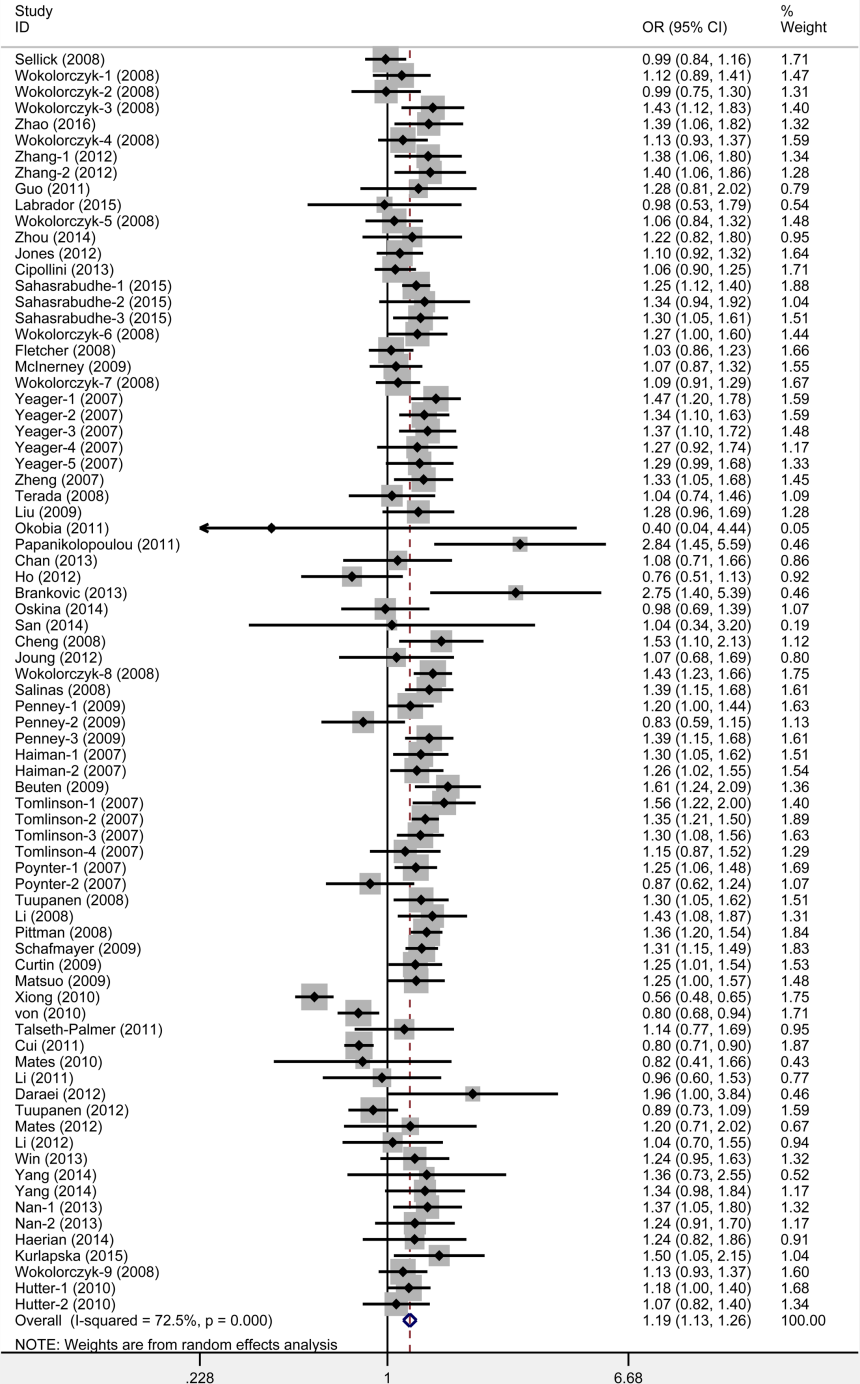
Meta-analysis for the association between rs6983267 polymorphism and cancer risk (dominant model: GG+GT vs. TT)

**Figure 3:**
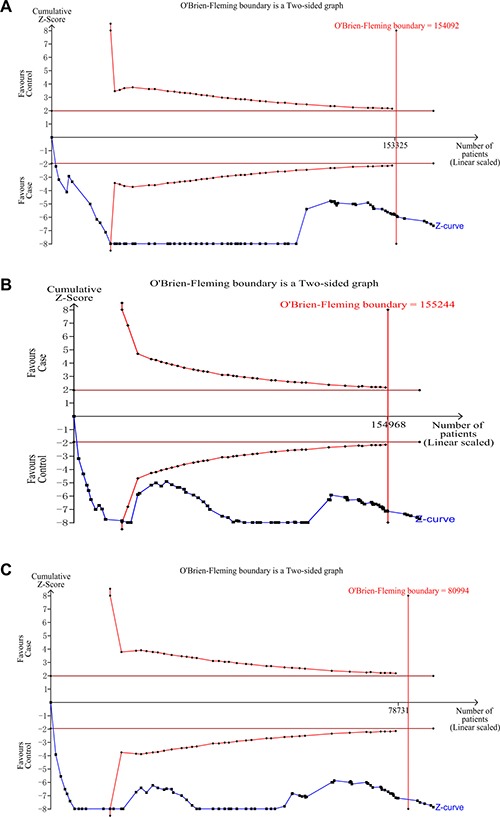
Trial sequential analysis of the association between rs6983267 polymorphism and cancer risk (**A**) dominant model; (**B**) recessive model; (**C**) homozygous model.

The main meta-analysis results of the association between rs6983267 polymorphism and cancer risk are shown in Table 1. We found that rs6983267 polymorphism significantly increased cancer risk in all five genetic models: dominant (GG+GT vs. TT, OR = 1.19, 95% CI = 1.13–1.26), recessive (GG vs. GT+TT, OR = 1.19, 95% CI = 1.14–1.25), homozygote (GG vs. TT, OR = 1.31, 95% CI = 1.23–1.40), heterozygous (GG vs. GT, OR = 1.14, 95% CI = 1.10–1.19) and allele (G vs. T, OR = 1.14, 95% CI = 1.11–1.18) models (Figure 2). False-positive report probability (FPRP) values for all significant findings at different prior probability levels are summarized in Supplementary Table 2. FPR*P* values at pre-specified prior probability of 0.01 were all lower than 0.2, indicating that the association between rs6983267 polymorphism and cancer risk was noteworthy. Outcomes of trial sequential analysis (TSA) were concordant with our results and revealed that rs6983267 polymorphism was significantly associated with cancer risk. Moreover, it also revealed that enough number of samples were included in this meta-analysis to reach a concrete conclusion as the cumulative Z-curve surpassed the O’Brien-Fleming boundary (Figure 3).

**Table 1:** Associations between rs6983267 polymorphism and cancer risk

Variables	*N*	GG+GT vs. TT	GG vs. GT+TT	GG vs. TT	GG vs. GT	G vs. T
		OR (95%CI)/*I*^2^%/P_*Q*_	OR (95%CI)/*I*^2^%/P_*Q*_	OR (95%CI)/*I*^2^%/P_*Q*_	OR (95%CI)/*I*^2^%/P_*Q*_	OR (95%CI)/*I*^2^%/P_*Q*_
**Overall**	78	**1.19 (1.13, 1.26)/7s3/<10**^−3^	**1.19 (1.14, 1.25)/68/<10**^−3^	**1.31 (1.23, 1.40)/76/<10**^−3^	**1.14 (1.10, 1.19)/53/<10**^−3^	**1.14 (1.11, 1.18)/76/<10**^−3^
**Ethnicity**						
Caucasian	57	**1.22 (1.17, 1.28)/53/<10**^−3^	**1.21 (1.16, 1.26)/51/<10**^−3^	**1.34 (1.26, 1.42)/63/<10**^−3^	**1.15 (1.11, 1.19)/30/0.019**	**1.16 (1.12, 1.19)/62/<10**^−3^
Asian	18	1.14 (0.96, 1.34)/85/<10^−3^	**1.19 (1.03, 1.37)/79/<10**^−3^	**1.26 (1.03, 1.55)/84/<10**^−3^	1.14 (0.99, 1.31)/75/<10^−3^	**1.12 (1.02, 1.24)/84/<10**^−3^
African	3	1.05 (0.74, 1.50)/71/0.031	1.03 (0.93, 1.13)/0/0.667	1.05 (0.76, 1.45)/60/0.082	1.03 (0.93, 1.13)/0/0.632	1.03 (0.96, 1.10)/38/0.198
**Cancer type**						
Colorectal cancer	32	**1.15 (1.04, 1.27)/84/<10**^−3^	**1.17 (1.09, 1.26)/78/<10**^−3^	**1.26 (1.11, 1.42)/85/<10**^−3^	**1.13 (1.05, 1.20)/68/<10**^−3^	**1.12 (1.06, 1.19)/85/<10**^−3^
Prostate cancer	25	**1.29 (1.21, 1.39)/39/0.025**	**1.31 (1.25, 1.38)/8/0.348**	**1.50 (1.38, 1.64)/37/0.034**	**1.23 (1.17, 1.30)/0/0.674**	**1.22 (1.17, 1.27)/32/0.063**
Thyroid cancer	6	**1.20 (1.12, 1.29)/0/0.468**	**1.17 (1.04, 1.31)/54/0.056**	**1.29 (1.18, 1.41)/44/0.109**	**1.11 (1.00, 1.25)/47/0.096**	**1.14 (1.06, 1.21)/48/0.088**
Gastric cancer	4	1.11 (0.93, 1.32)/0/0.818	0.90 (0.67, 1.22)/58/0.069	1.02 (0.82, 1.26)/0/0.742	0.86 (0.61, 1.23)/65/0.035	1.01 (0.91, 1.12)/0/0.610
Lung cancer	3	**1.25 (1.09, 1.44)/8/0.338**	**1.21 (1.05, 1.40)/0/0.617**	**1.36 (1.15, 1.62)/4/0.352**	1.13 (0.97, 1.32)/0/0.896	**1.17 (1.07, 1.28)/22/0.279**
Breast cancer	3	1.06 (0.95, 1.18)/0/0.907	1.05 (0.95, 1.17)/0/0.510	1.09 (0.96, 1.24)/0/0.624	1.03 (0.93, 1.16)/0/0.561	1.04 (0.98, 1.11)/0/0.633
Other cancer	5	1.15 (0.99, 1.35)/57/0.054	**1.12 (1.01, 1.23)/18/0.298**	**1.24 (1.02, 1.51)/59/0.047**	1.09 (0.98, 1.20)/0/0.658	**1.11 (1.01, 1.22)/57/0.053**
**Study quality**						
High (≥ 9)	52	**1.26 (1.20, 1.31)/46/<10**^−3^	**1.24 (1.19, 1.29)/51/<10**^−3^	**1.40 (1.32, 1.48)/58/<10**^−3^	**1.17 (1.13, 1.22)/35/0.007**	**1.18 (1.15, 1.22)/59/<10**^−3^
Low (< 9)	26	1.06 (0.94, 1.18)/76/<10^−3^	1.07 (0.99, 1.15)/56/<10^−3^	1.10 (0.97, 1.24)/71/<10^−3^	1.06 (0.98, 1.14)/52/0.001	1.03 (0.97, 1.11)/69/<10^−3^
**Source of controls**						
PB	41	**1.20 (1.13, 1.27)/70/<10**^−3^	**1.19 (1.12, 1.26)/75/<10**^−3^	**1.31 (1.20, 1.42)/79/<10**^−3^	**1.13 (1.08, 1.19)/59/<10**^−3^	**1.14 (1.10, 1.19)/80/<10**^−3^
HB	37	**1.19 (1.08, 1.31)/75/<10**^−3^	**1.20 (1.12, 1.29)/57/<10**^−3^	**1.32 (1.17, 1.49)/74/<10**^−3^	**1.15 (1.08, 1.23)/45/0.002**	**1.15 (1.09, 1.21)/71/<10**^−3^
**Genotyping method**						
PCR-RFLP	28	**1.15 (1.04, 1.28)/81/<10**^−3^	**1.18 (1.10, 1.26)/60/<10**^−3^	**1.25 (1.11, 1.42)/79/<10**^−3^	**1.15 (1.08, 1.22)/45/0.006**	**1.12 (1.06, 1.19)/78/<10**^−3^
TaqMan	15	**1.18 (1.03, 1.34)/80/<10**^−3^	**1.16 (1.01, 1.33)/84/<10**^−3^	**1.28 (1.05, 1.57)/87/<10**^−3^	1.08 (0.97, 1.21)/72/<10^−3^	**1.13 (1.02, 1.25)/88/<10**^−3^
Other methods	35	**1.28 (1.23, 1.33)/0/0.548**	**1.23 (1.18, 1.27)/18/0.183**	**1.40 (1.34, 1.47)/18/0.175**	**1.16 (1.11, 1.20)/7/0.348**	**1.18 (1.16, 1.21)/18/0.179**

HB: hospital-based controls; PB: publication-based controls; OR: Odds ratio; CI: Confidence interval; *P_Q_*: *P* value of the *Q*-test for heterogeneity test. Bold values are significant associations before the FPRP analyses.

### Subgroup analyses

**Figure 4:**
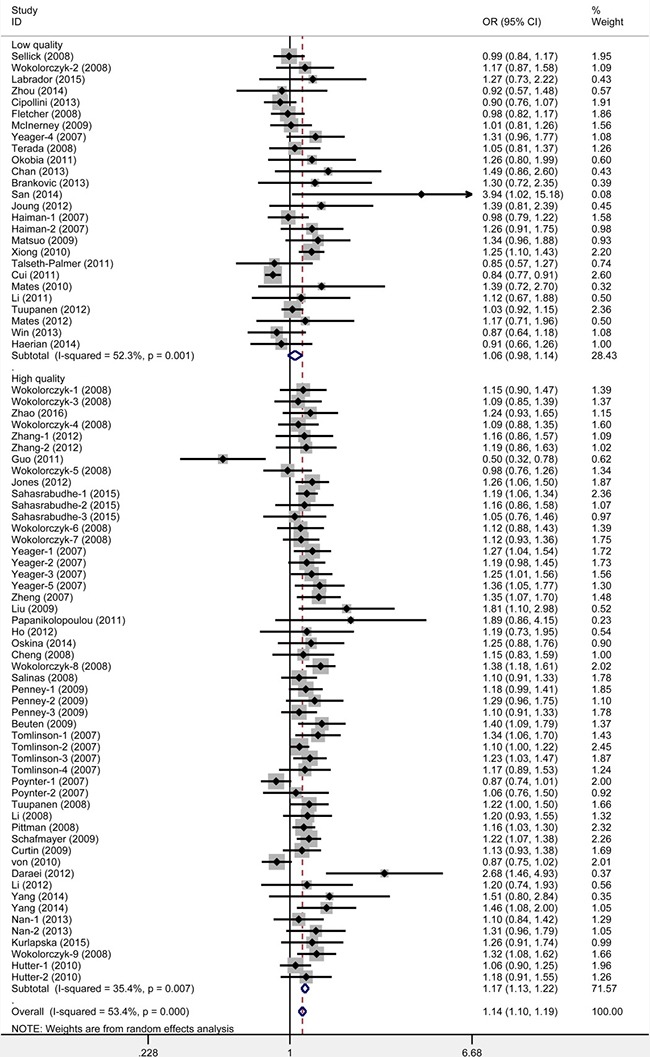
Meta-analysis for the association between rs6983267 polymorphism and cancer risk: subgroup analysis by quality appraisal score (heterozygote model: GG vs. GT)

When studies were stratified under source of controls and genotyping method, significant results were detected in all subgroups (Table 1). Moreover, when studies were stratified by quality score, an increased cancer risk was observed in high quality subgroup (Figure 4). With the assumption of prior probability of 0.01, these statistically significant associations were noteworthy (FPR*P* value < 0.2) for population-based, hospital-based and high quality subgroups under all five models (Supplementary Table 2), and for PCR-RFLP subgroup under recessive, homozygote, heterozygous and allele models.

When studies were stratified in ethnicity, significant associations were found in Caucasians and Asians, but not in Africans (Table 1). Moreover, when studies were stratified in to cancer type, significant associations were found in colorectal cancer, prostate cancer, thyroid cancer, lung cancer and other cancers subgroups, but not in gastric cancer and breast cancer. FPRP analyses suggested that these statistically significant associations were noteworthy for Caucasians, colorectal cancer, prostate cancer, thyroid cancer and lung cancer (Supplementary Table 2).

Furthermore, stratified analyses revealed a significant association between rs6983267 polymorphism and risk of colorectal cancer among Caucasians (Table 2). FPRP analyses suggested that this positive association was noteworthy under all five models (FPRP range: 0.000–0.007, Table 3). For prostate cancer, increased risks were observed among Caucasians, Asians and Africans (Table 2). FPRP analyses indicated that these associations were noteworthy for Caucasians (FPR*P* < 0.001) under all five models and Asians (FPRP range: 0.026–0.194) under recessive model, homozygote model and allele model (Table 3). For thyroid cancer, increased risks were revealed among Caucasians and Asians (Table 2). FPRP analyses suggested that these associations were noteworthy for Caucasians (FPRP range: 0.000–0.182) under dominant model, homozygote model and allele model (Table 3). For lung cancer, increased risk was found among Asians (Table 2). FPRP analyses suggested that this positive association was noteworthy under dominant model, homozygote model and allele model (FPRP range: 0.015–0.079, Table 3).

**Table 2:** Stratified analyses of rs6983267 polymorphism on cancer risk by cancer type and ethnicity

Variables	*N*	GG+GT vs. TT	GG vs. GT+TT	GG vs. TT	GG vs. GT	G vs. T
		OR (95%CI)/*I*^2^%/*P_Q_*	OR (95%CI)/*I*^2^%/*P_Q_*	OR (95%CI)/*I*^2^%/*P_Q_*	OR (95%CI)/*I*^2^%/*P_Q_*	OR (95%CI)/*I*^2^%/*P_Q_*
**Colorectal cancer**	32	1.15 (1.04, 1.27)/84/<10^−3^	1.17 (1.09, 1.26)/78/<10^−3^	1.26 (1.11, 1.42)/85/<10^−3^	1.13 (1.05, 1.20)/68/<10^−3^	1.12 (1.06, 1.19)/85/<10^−3^
Caucasian	24	**1.23 (1.15, 1.32)/52/0.002**	**1.19 (1.12, 1.27)/58/<10**^−3^	**1.33 (1.21, 1.46)/65/<10**^−3^	**1.13 (1.07, 1.20)/42/0.016**	**1.15 (1.10, 1.21)/65/<10**^−3^
Asian	7	0.98 (0.75, 1.30)/88/<10^−3^	1.15 (0.92, 1.44)/85/<10^−3^	1.10 (0.80, 1.49)/86/<10^−3^	1.17 (0.92, 1.49)/86/<10^−3^	1.05 (0.91, 1.22)/86/<10^−3^
African	1	0.89 (0.73, 1.09)	1.00 (0.90, 1.12)	0.90 (0.74, 1.11)	1.03 (0.92, 1.15)	0.98 (0.90, 1.07)
**Prostate cancer**	25	1.29 (1.21, 1.39)/39/0.025	1.31 (1.25, 1.38)/8/0.348	1.50 (1.38, 1.64)/37/0.034	1.23 (1.17, 1.30)/0/0.674	1.22 (1.17, 1.27)/32/0.063
Caucasian	18	**1.32 (1.21, 1.45)/51/0.007**	**1.33 (1.26, 1.41)/0/0.530**	**1.54 (1.40, 1.70)/43/0.027**	**1.25 (1.18, 1.32)/0/0.821**	**1.24 (1.18, 1.30)/42/0.034**
Asian	5	**1.19 (1.04, 1.36)/0/0.820**	**1.29 (1.10, 1.52)/29/0.227**	**1.41 (1.17, 1.72)/14/0.325**	**1.26 (1.06, 1.49)/5/0.379**	**1.18 (1.07, 1.29)/0/0.523**
African	2	**1.29 (1.04, 1.60)/0/0.340**	1.09 (0.91, 1.31)/0/0.612	1.27 (0.98, 1.64)/0/0.356	1.03 (0.85, 1.25)/0/0.338	**1.13 (1.00, 1.27)/0/0.906**
**Thyroid cancer**	6	1.20 (1.12, 1.29)/0/0.468	1.17 (1.04, 1.31)/54/0.056	1.29 (1.18, 1.41)/44/0.109	1.11 (1.00, 1.25)/47/0.096	1.14 (1.06, 1.21)/48/0.088
Caucasian	5	**1.19 (1.10, 1.28)/1/0.402**	**1.17 (1.03, 1.33)/63/0.029**	**1.27 (1.10, 1.48)/55/0.063**	1.12 (0.99, 1.27)/56/0.057	**1.13 (1.05, 1.22)/56/0.058**
Asian	1	**1.30 (1.05, 1.61)**	1.19 (0.87, 1.62)	1.35 (0.97, 1.88)	1.05 (0.76, 1.46)	**1.20 (1.03, 1.40)**
**Lung cancer**	3	1.25 (1.09, 1.44)/8/0.338	1.21 (1.05, 1.40)/0/0.617	1.36 (1.15, 1.62)/4/0.352	1.13 (0.97, 1.32)/0/0.896	1.17 (1.07, 1.28)/22/0.279
Caucasian	1	1.13 (0.93, 1.37)	1.13 (0.92, 1.38)	1.20 (0.94, 1.53)	1.09 (0.88, 1.35)	1.09 (0.97, 1.23)
Asian	2	**1.39 (1.15, 1.68)/0/0.934**	**1.30 (1.06, 1.59)/0/0.896**	**1.55 (1.21, 1.97)/0/0.922**	1.17 (1.94, 1.46)/0/0.931	**1.26 (1.11, 1.42)/0/0.845**

OR: Odds ratio; CI: Confidence interval; *P_Q_*: *P* value of the *Q*-test for heterogeneity test. Bold values are significant associations before the FPRP analyses.

**Table 3:** False-positive report probability values for associations between the rs6983267 polymorphism and cancer risk

Significant association	OR (95%CI)	*P* ^a^	Statistical power ^b^	Prior probability				
				0.25	0.1	0.01	0.001	0.0001
**Colorectal cancer - Caucasian**								
GG+GT *vs*. TT	1.23 (1.15, 1.32)	< 0.001	1.000	**0.000**	**0.000**	**0.000**	**0.000**	**0.000**
GG *vs*. GT+TT	1.19 (1.12, 1.27)	< 0.001	1.000	**0.000**	**0.000**	**0.000**	**0.000**	**0.002**
GG *vs*. TT	1.33 (1.21, 1.46)	< 0.001	0.994	**0.000**	**0.000**	**0.000**	**0.000**	**0.000**
GG *vs*. GT	1.13 (1.07, 1.20)	< 0.001	1.000	**0.000**	**0.001**	**0.007**	**0.063**	0.402
G *vs*. T	1.15 (1.10, 1.21)	< 0.001	1.000	**0.000**	**0.000**	**0.000**	**0.000**	**0.001**
**Prostate cancer - Caucasian**								
GG+GT *vs*. TT	1.32 (1.21, 1.45)	< 0.001	0.996	**0.000**	**0.000**	**0.000**	**0.000**	**0.000**
GG *vs*. GT+TT	1.33 (1.26, 1.41)	< 0.001	1.000	**0.000**	**0.000**	**0.000**	**0.000**	**0.000**
GG *vs*. TT	1.54 (1.40, 1.70)	< 0.001	0.313	**0.000**	**0.000**	**0.000**	**0.000**	**0.000**
GG *vs*. GT	1.25 (1.18, 1.32)	< 0.001	1.000	**0.000**	**0.000**	**0.000**	**0.000**	**0.000**
G *vs*. T	1.24 (1.18, 1.30)	< 0.001	1.000	**0.000**	**0.000**	**0.000**	**0.000**	**0.000**
**Prostate cancer - Asian**								
GG+GT vs. TT	1.19 (1.04, 1.36)	0.011	1.000	**0.031**	**0.088**	0.514	0.914	0.991
GG vs. GT+TT	1.29 (1.10, 1.52)	0.002	0.964	**0.007**	**0.021**	**0.194**	0.709	0.961
GG vs. TT	1.41 (1.17, 1.72)	0.001	0.729	**0.003**	**0.009**	**0.087**	0.490	0.906
GG vs. GT	1.26 (1.06, 1.49)	0.007	0.979	**0.021**	**0.060**	0.411	0.876	0.986
G *vs*. T	1.18 (1.07, 1.29)	0.001	1.000	**0.001**	**0.002**	**0.026**	0.214	0.732
**Prostate cancer** - **African**								
GG+GT *vs*. TT	1.29 (1.04, 1.60)	0.020	0.915	**0.063**	**0.168**	0.689	0.957	0.996
G *vs*. T	1.13 (1.00, 1.27)	0.040	1.000	**0.108**	0.266	0.799	0.976	0.998
**Thyroid cancer - Caucasian**								
GG+GT *vs*. TT	1.19 (1.10, 1.28)	< 0.001	1.000	**0.000**	**0.000**	**0.000**	**0.003**	**0.028**
GG *vs*. GT+TT	1.17 (1.03, 1.33)	0.016	1.000	**0.047**	**0.128**	0.618	0.942	0.994
GG *vs*. TT	1.27 (1.10, 1.48)	0.002	0.983	**0.007**	**0.020**	**0.182**	0.691	0.957
G *vs*. T	1.13 (1.05, 1.22)	0.002	1.000	**0.005**	**0.016**	**0.149**	0.639	0.947
**Thyroid cancer -** **Asian**								
GG+GT *vs*. TT	1.30 (1.05, 1.61)	0.016	0.905	**0.051**	**0.139**	0.639	0.947	0.994
G *vs*. T	1.20 (1.03, 1.40)	0.020	0.998	**0.058**	**0.156**	0.670	0.953	0.995
**Lung cancer - Asian**								
GG+GT *vs*. TT	1.39 (1.15, 1.68)	< 0.001	0.785	**0.003**	**0.008**	**0.077**	0.456	0.894
GG *vs*. GT+TT	1.30 (1.06, 1.59)	0.011	0.918	**0.034**	**0.095**	0.535	0.921	0.991
GG *vs*. TT	1.55 (1.21, 1.97)	< 0.001	0.394	**0.003**	**0.008**	**0.079**	0.463	0.896
G *vs*. T	1.26 (1.11, 1.42)	< 0.001	0.998	**0.000**	**0.001**	**0.015**	**0.131**	0.602

The results in false-positive report probability analysis were in bold, if the prior probability < 0.2. OR: odds ratio; CI: confidence interval; ^a^ *P* value for significant test; ^b^ Statistical power was calculated using the number of observations in the meta-analysis and the OR and *P* value in this table.

### Heterogeneity analyses

**Figure 5:**
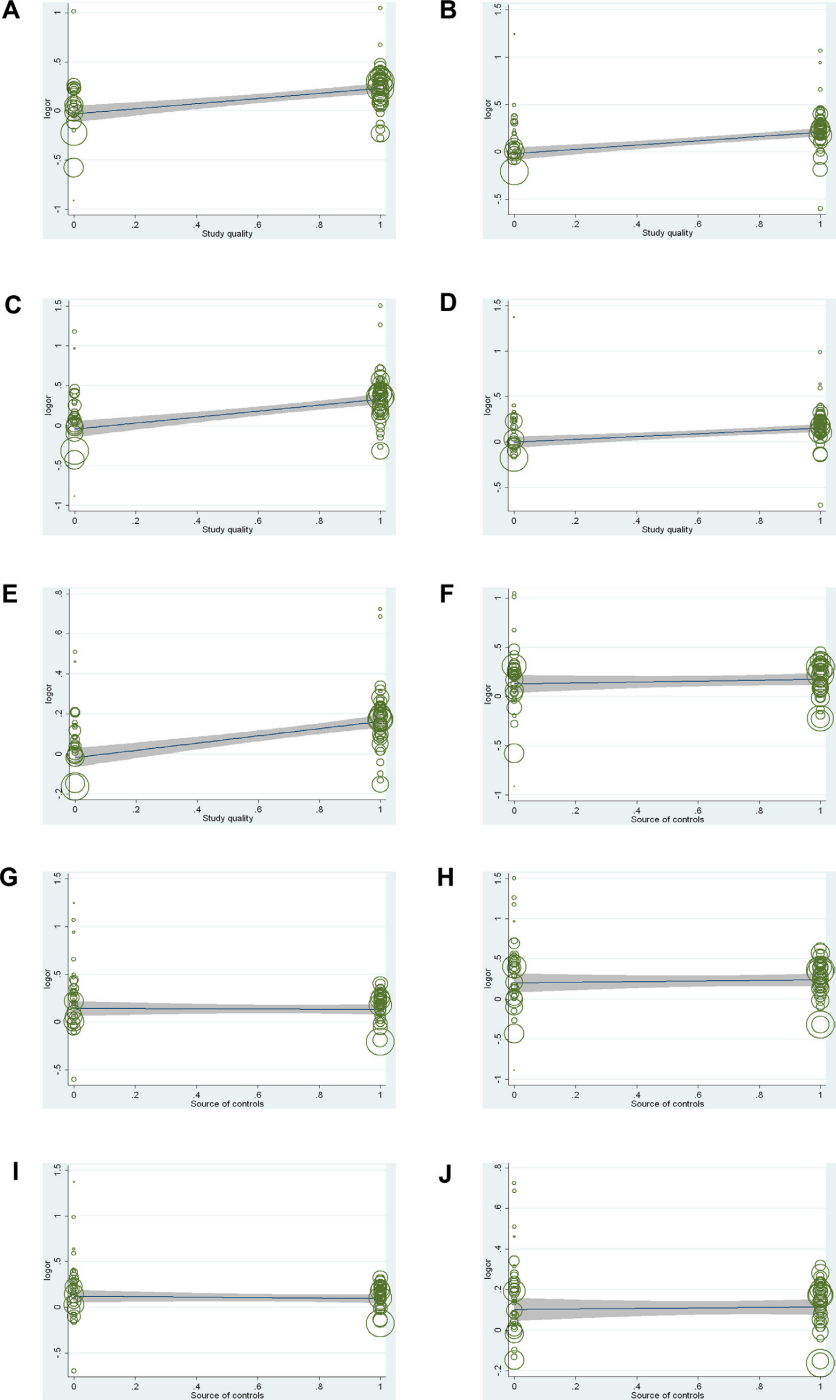
Meta-regression analysis of the main characteristics of the 78 studies Meta-regression analysis of study quality (**A**) dominant model; (**B**) recessive model; (**C**) homozygote model; (**D**) heterozygote model; (**E**) allele model) and source of controls (**F**) dominant model; (**G**) recessive model; (**H**) homozygote model; (**I**) heterozygote model; (**J**) allele model).

Q test and I^2^ statistics were applied to evaluate the heterogeneity during our study. There was significant heterogeneity observed in the overall analysis. Therefore, we conducted meta-regression to explore the source of heterogeneity by ethnicity, cancer type, genotyping method, study quality and source of controls. As shown in Figure 5 and Supplementary Table 3, cancer type (heterozygote model: *P* = 0.043, recessive model: *P* = 0.020) and study quality (allelic model: *P* < 0.001, homozygote model: *P* < 0.001, heterozygote model: *P* = 0.003, recessive model: *P* < 0.001, dominant model: *P* < 0.001) were factors that contributed to the observed heterogeneity across all studies. However, combining with these two factors could explain only 33.89% (heterozygote model) or 49.57% (recessive model) of the τ^2^ value, indicating that cancer type and study quality could explain one part of the heterogeneity. Otherwise, ethnicity, genotyping method and source of controls did not contribute the heterogeneity across the overall studies (*P* > 0.05, Supplementary Table 3).

### Publication bias

**Figure 6:**
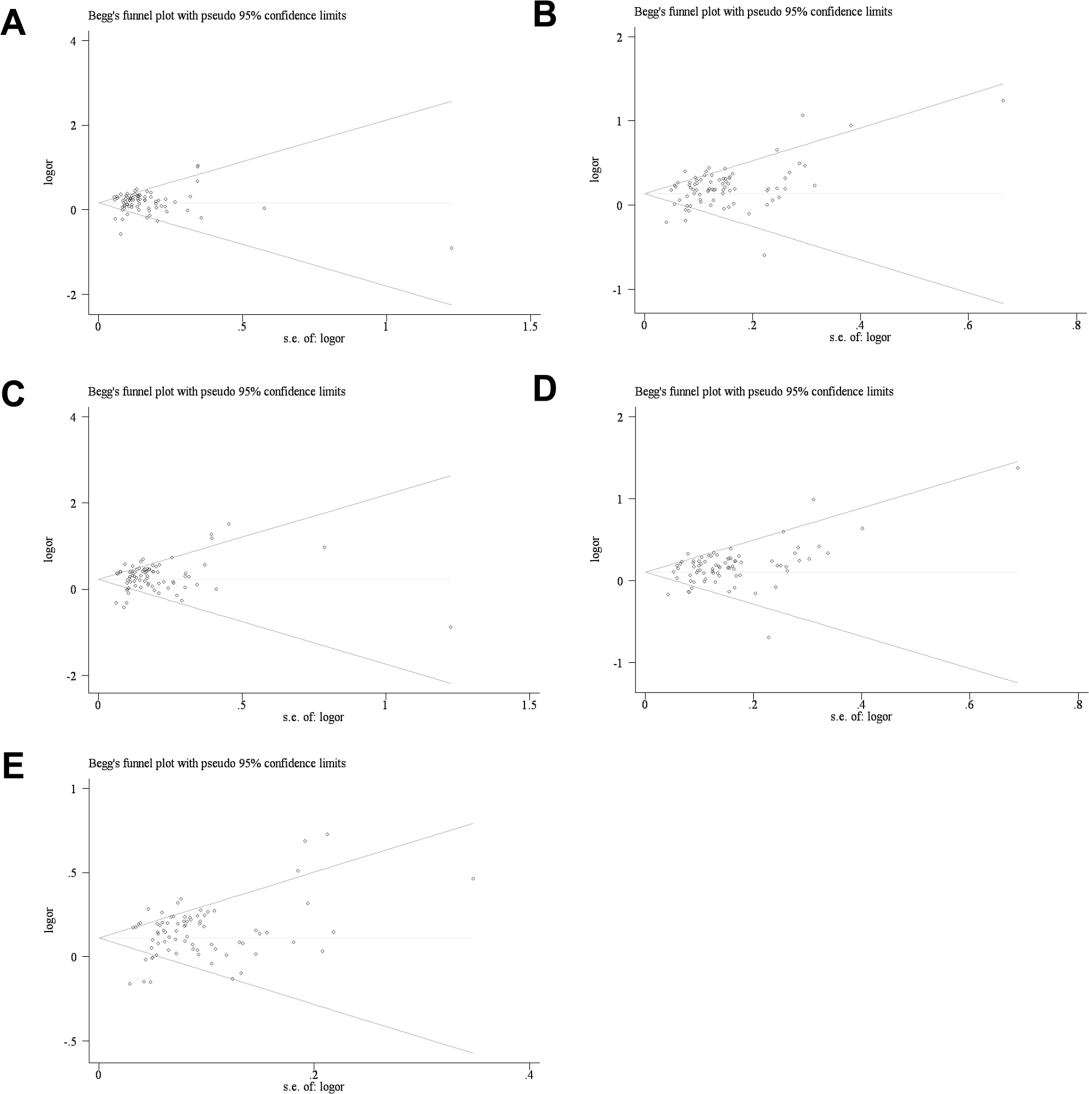
Begg's test for publication bias (**A**) dominant model; (**B**) recessive model; (**C**) homozygous model; (**D**) heterozygous model; (**E**) allele model.

Begg's and Egger's tests were performed to assess the publication bias. The shape of the Begg's funnel plots seemed symmetrical (Figure 6). Meanwhile, Egger's test suggested that there is no evidence of significant publication bias (*P*_Egger_=0.100 for dominant model, *P*_Egger_= 0.944 for recessive model, *P*_Egger_= 0.233 for homozygote model, *P*_Egger_=0.692 for heterozygote model, and *P*_Egger_= 0.484 for allele model) in this meta-analysis.

### Sensitivity analysis

**Figure 7:**
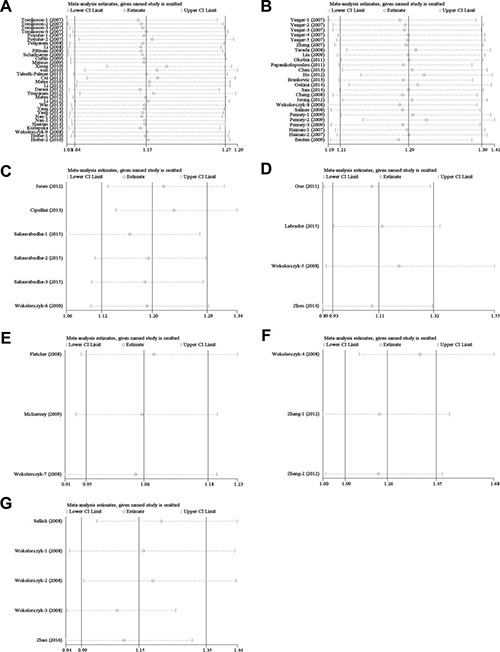
Sensitivity analyses of the studies (**A**) colorectal cancer; (**B**) prostate cancer; (**C**) thyroid cancer; (**D**) gastric cancer; (**E**) breast cancer; (**F**) lung cancer; (**G**) other cancer.

To evaluate the influence of individual study on the pooled ORs and 95% CIs, we excluded one study at each time. Results indicated that none of single study substantially changed the corresponding pooled ORs and 95% CIs (Figure 7), and demonstrated that our meta-analysis was relatively stable and credible.

## DISCUSSION

It has been well established that genetics determine the risk of cancer in the last decades [90]. Since SNP is the main cause of human genetic variation, the connection between SNP and individual risk of cancer has drawn considerable attention. Recently, epidemiological studies evaluated the relationship between rs6983267 polymorphism and risk of multiple cancer types, while the results were inconsistent. In previous meta-analyses [15–18, 20, 91, 92] , the limited number of studies that included or not exclusion of studies that were not in HWE, then the validity of conclusions may decrease. Moreover, many relevant case-control studies, including more cancer types, were published [40, 89], while these articles have not been discussed in previous meta-analyses. Hence, to provide a good comprehensive conclusion, a meta-analysis of all available studies was conducted.

We performed a meta-analysis of 78 case-control studies from 54 articles (73,996 cases and 96,741 controls) to clarify the relationship between rs6983267 polymorphism and cancer susceptibility. Significant associations between rs6983267 polymorphism and cancer susceptibility were found under most of assumed comparisons, either in overall or in stratified analyses by ethnicity, cancer type, study quality, source of controls and genotyping method. When studies were stratified by study quality and source of controls, significant associations were observed under the assumed comparisons in the high quality subgroup, the publication-based subgroup and the hospital-based subgroup, but not in the low quality subgroup. Lack of association in the low quality subgroup was probably due to the fact that this group could not represent the general population sufficiently.

In addition, stratified analyses by ethnicity revealed a significant association between rs6983267 and colorectal cancer in Caucasians, but not in Asians and Africans. The rs6983267 was identified as a common susceptibility variant for colorectal cancer by three previous GWASs [8, 9, 93] in Caucasians, which was consistent with our study. However, our results were different than two previous meta-analyses [17, 18], which reported that there was a significant association between rs6983267 and colorectal cancer in Asians. Possible reasons for this difference could be explained as follows: 1) one study by *Hutter et al*. [61] was carried out in Caucasians, while it was incorporated into the Asian subgroup in a previous meta-analysis [17]; 2) we included 7 case-control studies in Asians, instead of only 4 studies in a meta-analysis by *Haerian et al*. [18], and therefore, results of our meta-analysis were more credible; 3) lack of further research in different stratified analyses prevented comprehensive understanding in a recent meta-analysis [21]; 4) our results were based on sufficient evidence, which was proved by FPRP for the first time.

Moreover, we also found that rs6983267 polymorphism was a risk factor for the susceptibility to prostate cancer in Caucasians and Asians, which was consistent with two independent GWASs [10, 11]. However, our outcomes were different to the results shown by *Ho et al*. [73], which demonstrated that rs6983267 polymorphism was not associated with prostate cancer. This discrepancy may be caused by the limited sample size. *Ho et al*. [73] included only 521 subjects (247 cases and 274 controls), which may lack sufficient power to support or deny an association. Previous meta-analyses also focused on the relationship between the rs6983267 and prostate cancer. However, our outcomes were different to previous meta-analyses [16, 91], which indicated that rs6983267 polymorphism was associated with prostate cancer risk in Caucasians but not in Asians. Possible reasons for this difference could be explained as follows: 1) this discrepancy may be come from the limited sample size. For example, *Yang et al*. [16] included only 3 studies (805 cases and 703 controls) in Asians, whereas we included 5 studies (2200 cases and 1864 controls); 2) we excluded the studies that do not follow HWE, however, *Li et al*. [91] did not. In addition, when compared with the meta-analysis by *Zhu et al*. [15], although we reached the same conclusion, our analysis has some advantages: 1) the sample size of *Zhu et al*. was relatively small (16,753 cases and 14,802 controls); 2) to avoid false positive findings, FPRP analyses were performed for all significant findings observed in our study.

Furthermore, we found that rs6983267 conferred a higher thyroid cancer risk among Caucasians than Asians. It was partially consistent with the consequence of the meta-analysis by *Li et al*. [20], while the sample size in our study was much more times elevated than theirs. Similarly, we also found that rs6983267 conferred a higher lung cancer risk among Asians, and FPRP analyses suggested that this positive association was noteworthy.

Moderate heterogeneity between eligible studies was identified for all genetic models in the overall comparisons. Common reasons of heterogeneity may include differences in sample selection (e.g., source of controls, HWE) or studied populations (e.g., geographic location), or methods (e.g., genotyping method), or other factors (e.g., study quality and cancer type). Meta-regression analyses indicated that the potential sources of heterogeneity were cancer type and study quality. Nevertheless, when studies were stratified by cancer type and study quality, heterogeneity was still high in the colorectal cancer subgroup, the high quality subgroup and the low quality subgroup. These two analyses provided evidence that heterogeneity might also be explained by other confounding factors. In general, more uniform and rigorous studies were required.

Since quite many false positive results were found in those association studies between genetic variants and complex diseases due to the widely use of significance threshold (*P* < 0.05) [94]. To avoid false positive findings, our meta-analysis adopted FPRP analysis, which is based not only on the observed *P* value but also on the prior probability of hypothesis, making our results more reliable [95].

In the current study, there existed several advantages: 1) more studies were included in our meta-analysis; 2) more comprehensive subgroup analyses were conducted, and significant associations were found when we restricted to the high quality subgroup and population-based controls subgroup; 3) our results were based on sufficient evidence, which were proved by TSA for the first time; 4) to avoid false positive findings, FPRP analyses were used for all significant findings observed in our study. However, some limitations should also be emphasized. First, in the subgroup analysis, we found that our analysis was limited on Caucasians, Asians and Africans, so we do not know whether these conclusions can also be adopted in other populations. Second, the number of included studies in some subgroups was relatively small, which might create significant or insignificant associations by chance due to insufficient statistical power. Third, this study is a summary of multiple data sources. Due to the lack of original data, we could not evaluate the cancer susceptibility stratified by drinking status, smoking, carcinogen, radiation exposure, and other risk factors. Thus, more studies by standardized unbiased methods are required to offer more detailed individual data.

In summary, this systematical meta-analysis indicated that rs6983267 polymorphism significantly increased the risk of colorectal cancer in Caucasians, prostate cancer in Caucasians and Asians, thyroid cancer in Caucasians and lung cancer in Asians. In addition, significant association between rs6983267 polymorphism and cancer risk was observed in the high quality subgroup, the publication-based subgroup, the hospital-based subgroup, and the PCR-RFLP subgroup. Further multi-center, large-cohort and well-designed studies are necessary to validate our findings.

## MATERIALS AND METHODS

### Identification of the eligible studies

We systematically searched on PubMed, EMBASE, Web of Science and China National Knowledge Infrastructure (CNKI) electronic databases for relevant studies published before November 1, 2016. A detailed search strategy is presented in Supplementary Table 4. If studies were performed with overlapping data, only the largest or the latest studies would be included. Two independent authors conducted the search. Finally, we also searched the reference lists of all retrieved articles for potential studies manually.

### Inclusion criteria

Enrolled studies should meet the following eligibility criteria: (1) case-control design; (2) investigating the association between rs6983267 polymorphism and cancer risk; (3) describing the genotype distributions in detail to calculate the OR and 95% CI in cases and controls; (4) observed genotype frequencies in controls must be consistent with Hardy-Weinberg Equilibrium (HWE).

### Exclusion criteria

The exclusion criteria were as follows: (1) not concerned with cancer risk; (2) case only studies; (3) non-cancer subject only studies; (4) duplicate publications; (5) conference abstracts.

### Data extraction

Two investigators (M.Z. and X.W.) independently screened and extracted data from all eligible studies, with any disagreement resolved by consensus. The following information was collected: first author's surname, year of publication, ethnicity, country of origin, cancer type, source of controls, genotyping method, numbers of cases and controls, *P*-value of HWE in controls.

### Quality score assessment

The quality of each study was independently assessed by two investigators (X.W. and C.L.) who used quality scoring criteria modified from previous studies (Supplementary Table 5) [96, 97]. The evaluation items were as follows: ascertainment of cancer case, representativeness of case, representativeness of control, control selection, genotyping examination, and total sample size. Quality scores ranged from 0 (worst) to 12 (best). Studies scoring higher than 9 points were classified as high quality.

### Statistical analysis

All statistical analyses were performed by STATA version 12.0 (STATA Corporation, College Station, Texas, USA). *P* < 0.05 was considered statistically significant. The strength of association between rs6983267 and cancer risk was estimated by OR with 95% CI. Z test was applied to confirm whether an association was statistically significant. We measured the association based on five different genetic models: dominant model (GG+GT vs. TT), recessive model (GG vs. GT+TT), homozygote model (GG vs. TT), heterozygote model (GG vs. GT), and allele model (G vs. T). Cochran *Q*-test and I^2^ statistics were used to assess the between-study heterogeneity. A random-effect model was used to assess pooled ORs when *I2* (%) > 50% or *P* (Q) < 0.10, otherwise, a fixed-effect model was selected. Additionally, meta-regression analyses were used to detect the main sources of heterogeneity in our meta-analysis. Stratification analyses were performed by ethnicity, cancer type, study quality, genotyping method and source of controls. Sensitivity analyses were performed to assess the stability of the results. Furthermore, publication bias was assessed by Begg's and Egger's funnel plots, with potential publication bias if *P* < 0.05 and the plot was asymmetrical [98]. For each statistically significant association, false positive report probability (FPRP) analysis was performed using the method reported by *Wacholder et al*. [95]. We calculated FPRP assuming a prior probability of 0.01 as previously proposed [99]. An FPRP cutoff value of 0.2 was used and only result with FPR*P* value less than 0.2 was referred as noteworthy [95].

### Trial sequential analysis (TSA)

A meta-analysis is prone to systematic errors (bias) or random errors (play of chance) due to dispersed data and repeated significance testing [100]. To obtain more comprehensive assessment, trial sequential analysis (TSA) (version 0.9; Copenhagen Trial Unit, Copenhagen, Denmark, 2011) was used to calculate required information size (number of samples) and to confirm statistical reliability of meta-analysis.

In our study, we calculated the required information size by setting an overall type-I error of 5% and type-II error of 10% (a power of 90%). TSA plotted a two-sided graph where blue line indicates cumulative Z-score, red straight lines show significance boundaries of the conventional meta-analysis, and red lines sloping inwards represent trial sequential monitoring boundaries with adjusted *P*-values.

## SUPPLEMENTARY MATERIALS TABLES









## Abbreviations

SNP: single nucleotide polymorphism
OR: odds ratio
95% CI: 95% confidence interval
MYC: myelocytomatosis
GWAS: Genome-wide association study
ENPP2: ectonucleotide pyrophosphatase/phosphodiesterase 2 gene
NOV: nephroblastoma overexpressed gene
CNKI: China National Knowledge infrastructure
HWE: Hardy-Weinberg Equilibrium
HB: hospital-based controls
PB: publication-based controls
FPRP: false-positive report probability
TSA: trial sequential analysis.

## References

[1] Torre LA, Bray F, Siegel RL, Ferlay J, Lortet-Tieulent J, Jemal A (2015). Global cancer statistics, 2012. CA Cancer J Clin.

[2] Zhang M, Wang Z, Obazee O, Jia J, Childs EJ, Hoskins J, Figlioli G, Mocci E, Collins I, Chung CC, Hautman C, Arslan AA, Beane-Freeman L (2016). Three new pancreatic cancer susceptibility signals identified on chromosomes 1q32.1, 5p15.33 and 8q24.21. Oncotarget.

[3] Panagopoulos I, Moller E, Collin A, Mertens F (2008). The POU5F1P1 pseudogene encodes a putative protein similar to POU5F1 isoform 1. Oncol Rep.

[4] Wasserman NF, Aneas I, Nobrega MA (2010). An 8q24 gene desert variant associated with prostate cancer risk confers differential in vivo activity to a MYC enhancer. Genome Res.

[5] Ahmadiyeh N, Pomerantz MM, Grisanzio C, Herman P, Jia L, Almendro V, He HH, Brown M, Liu XS, Davis M, Caswell JL, Beckwith CA, Hills A (2010). 8q24 prostate, breast, and colon cancer risk loci show tissue-specific long-range interaction with MYC. Proc Natl Acad Sci USA.

[6] Gaetano CG, Samadi N, Tomsig JL, Macdonald TL, Lynch KR, Brindley DN (2009). Inhibition of autotaxin production or activity blocks lysophosphatidylcholine-induced migration of human breast cancer and melanoma cells. Mol Carcinog.

[7] Nikolsky Y, Ekins S, Nikolskaya T, Bugrim A (2005). A novel method for generation of signature networks as biomarkers from complex high throughput data. Toxicol Lett.

[8] Tomlinson I, Webb E, Carvajal-Carmona L, Broderick P, Kemp Z, Spain S, Penegar S, Chandler I, Gorman M, Wood W, Barclay E, Lubbe S, Martin L (2007). A genome-wide association scan of tag SNPs identifies a susceptibility variant for colorectal cancer at 8q24.21. Nat Genet.

[9] Zanke BW, Greenwood CM, Rangrej J, Kustra R, Tenesa A, Farrington SM, Prendergast J, Olschwang S, Chiang T, Crowdy E, Ferretti V, Laflamme P, Sundararajan S (2007). Genome-wide association scan identifies a colorectal cancer susceptibility locus on chromosome 8q24. Nat Genet.

[10] Yeager M, Orr N, Hayes RB, Jacobs KB, Kraft P, Wacholder S, Minichiello MJ, Fearnhead P, Yu K, Chatterjee N, Wang Z, Welch R, Staats BJ (2007). Genome-wide association study of prostate cancer identifies a second risk locus at 8q24. Nat Genet.

[11] Al Olama AA, Kote-Jarai Z, Giles GG, Guy M, Morrison J, Severi G, Leongamornlert DA, Tymrakiewicz M, Jhavar S, Saunders E, Hopper JL, Southey MC, Muir KR (2009). Multiple loci on 8q24 associated with prostate cancer susceptibility. Nat Genet.

[12] Tuupanen S, Yan J, Turunen M, Gylfe AE, Kaasinen E, Li L, Eng C, Culver DA, Kalady MF, Pennison MJ, Pasche B, Manne U, de la Chapelle A (2012). Characterization of the colorectal cancer-associated enhancer MYC-335 at 8q24: the role of rs67491583. Cancer Genet.

[13] Sur IK, Hallikas O, Vaharautio A, Yan J, Turunen M, Enge M, Taipale M, Karhu A, Aaltonen LA, Taipale J (2012). Mice lacking a Myc enhancer that includes human SNP rs6983267 are resistant to intestinal tumors. Science.

[14] Troutman SM, Sissung TM, Cropp CD, Venzon DJ, Spencer SD, Adesunloye BA, Huang X, Karzai FH, Price DK, Figg WD (2012). Racial disparities in the association between variants on 8q24 and prostate cancer: a systematic review and meta-analysis. Oncologist.

[15] Zhu HS, Zhang JF, Zhou JD, Zhang MJ, Hu HX (2015). Association between the 8q24 rs6983267 T/G polymorphism and prostate cancer risk: a meta-analysis. Genet Mol Res.

[16] Yang Y, Wang W, Zhang L, Zhang S, Liu G, Yu Y, Liao M (2016). Association of single nucleotide polymorphism rs6983267 with the risk of prostate cancer. Oncotarget.

[17] Li L, Lv L, Liang Y, Shen X, Zhou S, Zhu J, Ma R (2015). Association of 8q23-24 region (8q23.3 loci and 8q24.21 loci) with susceptibility to colorectal cancer: a systematic and updated meta-analysis. Int J Clin Exp Med.

[18] Haerian MS, Baum L, Haerian BS (2011). Association of 8q24.21 loci with the risk of colorectal cancer: a systematic review and meta-analysis. J Gastroenterol Hepatol.

[19] Yao K, Hua L, Wei L, Meng J, Hu J (2015). Correlation Between CASC8, SMAD7 Polymorphisms and the Susceptibility to Colorectal Cancer: An Updated Meta-Analysis Based on GWAS Results. Medicine (Baltimore).

[20] Li J, Wang X, Dong J (2016). Association of rs6983267 Polymorphism and Thyroid Cancer Susceptibility: A Systematic Review and Meta-Analysis. Med Sci Monit.

[21] Hong Y, Wu G, Li W, Liu D, He K (2016). A comprehensive meta-analysis of genetic associations between five key SNPs and colorectal cancer risk. Oncotarget.

[22] Berndt SI, Potter JD, Hazra A, Yeager M, Thomas G, Makar KW, Welch R, Cross AJ, Huang WY, Schoen RE, Giovannucci E, Chan AT, Chanock SJ (2008). Pooled analysis of genetic variation at chromosome 8q24 and colorectal neoplasia risk. Hum Mol Genet.

[23] Cussenot O, Azzouzi AR, Bantsimba-Malanda G, Gaffory C, Mangin P, Cormier L, Fournier G, Valeri A, Jouffe L, Roupret M, Fromont G, Sibony M, Comperat E (2008). Effect of genetic variability within 8q24 on aggressiveness patterns at diagnosis and familial status of prostate cancer. Clin Cancer Res.

[24] Ghoussaini M, Song H, Koessler T, Al Olama AA, Kote-Jarai Z, Driver KE, Pooley KA, Ramus SJ, Kjaer SK, Hogdall E, DiCioccio RA, Whittemore AS, Gayther SA (2008). Multiple loci with different cancer specificities within the 8q24 gene desert. J Natl Cancer Inst.

[25] Cicek MS, Slager SL, Achenbach SJ, French AJ, Blair HE, Fink SR, Foster NR, Kabat BF, Halling KC, Cunningham JM, Cerhan JR, Jenkins RB, Boardman LA (2009). Functional and clinical significance of variants localized to 8q24 in colon cancer. Cancer Epidemiol Biomarkers Prev.

[26] Bao BY, Pao JB, Lin VC, Huang CN, Chang TY, Lan YH, Lu TL, Lee HZ, Chen LM, Ting WC, Hsieh CJ, Huang SP (2010). Individual and cumulative association of prostate cancer susceptibility variants with clinicopathologic characteristics of the disease. Clin Chim Acta.

[27] Abuli A, Bessa X, Gonzalez JR, Ruiz-Ponte C, Caceres A, Munoz J, Gonzalo V, Balaguer F, Fernandez-Rozadilla C, Gonzalez D, de Castro L, Clofent J, Bujanda L (2010). Susceptibility genetic variants associated with colorectal cancer risk correlate with cancer phenotype. Gastroenterology.

[28] Murphy AB, Ukoli F, Freeman V, Bennett F, Aiken W, Tulloch T, Coard K, Angwafo F, Kittles RA (2012). 8q24 risk alleles in West African and Caribbean men. Prostate.

[29] Neta G, Yu CL, Brenner A, Gu F, Hutchinson A, Pfeiffer R, Sturgis EM, Xu L, Linet MS, Alexander BH, Chanock S, Sigurdson AJ (2012). Common genetic variants in the 8q24 region and risk of papillary thyroid cancer. Laryngoscope.

[30] Gerber MM, Hampel H, Schulz NP, Fernandez S, Wei L, Zhou XP, de la Chapelle A, Ewart Toland A (2012). Evaluation of allele-specific somatic changes of genome-wide association study susceptibility alleles in human colorectal cancers. PLoS One.

[31] Thean LF, Li HH, Teo YY, Koh WP, Yuan JM, Teoh ML, Koh PK, Tang CL, Cheah PY (2012). Association of Caucasian-identified variants with colorectal cancer risk in Singapore Chinese. PLoS One.

[32] Takatsuno Y, Mimori K, Yamamoto K, Sato T, Niida A, Inoue H, Imoto S, Kawano S, Yamaguchi R, Toh H, Iinuma H, Ishimaru S, Ishii H (2013). The rs6983267 SNP is associated with MYC transcription efficiency, which promotes progression and worsens prognosis of colorectal cancer. Ann Surg Oncol.

[33] Kupfer SS, Torres JB, Hooker S, Anderson JR, Skol AD, Ellis NA, Kittles RA (2009). Novel single nucleotide polymorphism associations with colorectal cancer on chromosome 8q24 in African and European Americans. Carcinogenesis.

[34] Tarleton HP, Chang SC, Park SL, Cai L, Ding B, He N, Hussain SK, Jiang Q, Mu LN, Rao J, Wang H, You NC, Yu SZ (2014). Genetic variation at 8q24, family history of cancer, and upper gastrointestinal cancers in a Chinese population. Fam Cancer.

[35] Akdi A, Perez G, Pastor S, Castell J, Biarnes J, Marcos R, Velazquez A (2011). Common variants of the thyroglobulin gene are associated with differentiated thyroid cancer risk. Thyroid.

[36] Zhang Y, Yi P, Chen W, Ming J, Zhu B, Li Z, Shen N, Shi W, Ke J, Zhao Q, Lu X, Xun X, Liu L (2014). Association between polymorphisms within the susceptibility region 8q24 and breast cancer in a Chinese population. Tumour Biol.

[37] Zhang Z, Wang JY, Wei D, Sun L, Wang XM, Zhang YG, Wang NN, Hui J, Zhang YR, Li XH, Zhang LH, Huo ZH, Jiao HY (2014). Association study of 4 single nucleotide polymorphisms in 8q24 region and prostate cancer. [Article in Chinese]. Journal of Ningxia Medical University.

[38] Ghazi S, von Holst S, Picelli S, Lindforss U, Tenesa A, Farrington SM, Campbell H, Dunlop MG, Papadogiannakis N, Lindblom A (2010). Colorectal cancer susceptibility loci in a population-based study: Associations with morphological parameters. Am J Pathol.

[39] Haiman CA, Le Marchand L, Yamamato J, Stram DO, Sheng X, Kolonel LN, Wu AH, Reich D, Henderson BE (2007). A common genetic risk factor for colorectal and prostate cancer. Nat Genet.

[40] Sellick GS, Broderick P, Fielding S, Catovsky D, Houlston RS (2008). Lack of a relationship between the common 8q24 variant rs6983267 and risk of chronic lymphocytic leukemia. Leukemia.

[41] Zheng SL, Sun J, Cheng Y, Li G, Hsu FC, Zhu Y, Chang BL, Liu W, Kim JW, Turner AR, Gielzak M, Yan G, Isaacs SD (2007). Association between two unlinked loci at 8q24 and prostate cancer risk among European Americans. J Natl Cancer Inst.

[42] Poynter JN, Figueiredo JC, Conti DV, Kennedy K, Gallinger S, Siegmund KD, Casey G, Thibodeau SN, Jenkins MA, Hopper JL, Byrnes GB, Baron JA, Goode EL (2007). Variants on 9p24 and 8q24 are associated with risk of colorectal cancer: results from the Colon Cancer Family Registry. Cancer Res.

[43] Tuupanen S, Niittymaki I, Nousiainen K, Vanharanta S, Mecklin JP, Nuorva K, Jarvinen H, Hautaniemi S, Karhu A, Aaltonen LA (2008). Allelic imbalance at rs6983267 suggests selection of the risk allele in somatic colorectal tumor evolution. Cancer Res.

[44] Cheng I, Plummer SJ, Jorgenson E, Liu X, Rybicki BA, Casey G, Witte JS (2008). 8q24 and prostate cancer: association with advanced disease and meta-analysis. Eur J Hum Genet.

[45] Li L, Plummer SJ, Thompson CL, Merkulova A, Acheson LS, Tucker TC, Casey G (2008). A common 8q24 variant and the risk of colon cancer: a population-based case-control study. Cancer Epidemiol Biomarkers Prev.

[46] Fletcher O, Johnson N, Gibson L, Coupland B, Fraser A, Leonard A, dos Santos Silva I, Ashworth A, Houlston R, Peto J (2008). Association of genetic variants at 8q24 with breast cancer risk. Cancer Epidemiol Biomarkers Prev.

[47] Pittman AM, Broderick P, Sullivan K, Fielding S, Webb E, Penegar S, Tomlinson I, Houlston RS (2008). CASP8 variants D302H and -652 6N ins/del do not influence the risk of colorectal cancer in the United Kingdom population. Br J Cancer.

[48] Salinas CA, Kwon E, Carlson CS, Koopmeiners JS, Feng Z, Karyadi DM, Ostrander EA, Stanford JL (2008). Multiple independent genetic variants in the 8q24 region are associated with prostate cancer risk. Cancer Epidemiol Biomarkers Prev.

[49] Terada N, Tsuchiya N, Ma Z, Shimizu Y, Kobayashi T, Nakamura E, Kamoto T, Habuchi T, Ogawa O (2008). Association of genetic polymorphisms at 8q24 with the risk of prostate cancer in a Japanese population. Prostate.

[50] Schafmayer C, Buch S, Volzke H, von Schonfels W, Egberts JH, Schniewind B, Brosch M, Ruether A, Franke A, Mathiak M, Sipos B, Henopp T, Catalcali J (2009). Investigation of the colorectal cancer susceptibility region on chromosome 8q24.21 in a large German case-control sample. Int J Cancer.

[51] McInerney N, Colleran G, Rowan A, Walther A, Barclay E, Spain S, Jones AM, Tuohy S, Curran C, Miller N, Kerin M, Tomlinson I, Sawyer E (2009). Low penetrance breast cancer predisposition SNPs are site specific. Breast Cancer Res Treat.

[52] Wokolorczyk D, Gliniewicz B, Sikorski A, Zlowocka E, Masojc B, Debniak T, Matyjasik J, Mierzejewski M, Medrek K, Oszutowska D, Suchy J, Gronwald J, Teodorczyk U (2008). A range of cancers is associated with the rs6983267 marker on chromosome 8. Cancer Res.

[53] Curtin K, Lin WY, George R, Katory M, Shorto J, Cannon-Albright LA, Bishop DT, Cox A, Camp NJ (2009). Meta association of colorectal cancer confirms risk alleles at 8q24 and 18q21. Cancer Epidemiol Biomarkers Prev.

[54] Penney KL, Salinas CA, Pomerantz M, Schumacher FR, Beckwith CA, Lee GS, Oh WK, Sartor O, Ostrander EA, Kurth T, Ma J, Mucci L, Stanford JL (2009). Evaluation of 8q24 and 17q risk loci and prostate cancer mortality. Clin Cancer Res.

[55] Beuten J, Gelfond JA, Martinez-Fierro ML, Weldon KS, Crandall AC, Rojas-Martinez A, Thompson IM, Leach RJ (2009). Association of chromosome 8q variants with prostate cancer risk in Caucasian and Hispanic men. Carcinogenesis.

[56] Liu M, Kurosaki T, Suzuki M, Enomoto Y, Nishimatsu H, Arai T, Sawabe M, Hosoi T, Homma Y, Kitamura T (2009). Significance of common variants on human chromosome 8q24 in relation to the risk of prostate cancer in native Japanese men. BMC Genet.

[57] Matsuo K, Suzuki T, Ito H, Hosono S, Kawase T, Watanabe M, Shitara K, Komori K, Kanemitsu Y, Hirai T, Yatabe Y, Tanaka H, Tajima K (2009). Association between an 8q24 locus and the risk of colorectal cancer in Japanese. BMC Cancer.

[58] Xiong F, Wu C, Bi X, Yu D, Huang L, Xu J, Zhang T, Zhai K, Chang J, Tan W, Cai J, Lin D (2010). Risk of genome-wide association study-identified genetic variants for colorectal cancer in a Chinese population. Cancer Epidemiol Biomarkers Prev.

[59] von Holst S, Picelli S, Edler D, Lenander C, Dalen J, Hjern F, Lundqvist N, Lindforss U, Pahlman L, Smedh K, Tornqvist A, Holm J, Janson M (2010). Association studies on 11 published colorectal cancer risk loci. Br J Cancer.

[60] Talseth-Palmer BA, Brenne IS, Ashton KA, Evans TJ, McPhillips M, Groombridge C, Suchy J, Kurzawski G, Spigelman A, Lubinski J, Scott RJ (2011). Colorectal cancer susceptibility loci on chromosome 8q23.3 and 11q23.1 as modifiers for disease expression in Lynch syndrome. J Med Genet.

[61] Hutter CM, Slattery ML, Duggan DJ, Muehling J, Curtin K, Hsu L, Beresford SA, Rajkovic A, Sarto GE, Marshall JR, Hammad N, Wallace R, Makar KW (2010). Characterization of the association between 8q24 and colon cancer: gene-environment exploration and meta-analysis. BMC Cancer.

[62] Cui R, Okada Y, Jang SG, Ku JL, Park JG, Kamatani Y, Hosono N, Tsunoda T, Kumar V, Tanikawa C, Kamatani N, Yamada R, Kubo M (2011). Common variant in 6q26-q27 is associated with distal colon cancer in an Asian population. Gut.

[63] Mates IN, Csiki I, Mates D, Constantinescu V, Badea P, Dinu D, Constantin A, Constantinoiu S (2010). Association of common genetic variants with colorectal cancer risk in a Romanian sample. Chirurgia (Bucur).

[64] Li M, Zhou Y, Chen P, Yang H, Yuan X, Tajima K, Cao J, Wang H (2011). Genetic variants on chromosome 8q24 and colorectal neoplasia risk: a case-control study in China and a meta-analysis of the published literature. PLoS One.

[65] Guo Y, Fang J, Liu Y, Sheng HH, Zhang XY, Chai HN, Jin W, Zhang KH, Yang CQ, Gao HJ (2011). Association between polymorphism rs6983267 and gastric cancer risk in Chinese population. World J Gastroenterol.

[66] Okobia MN, Zmuda JM, Ferrell RE, Patrick AL, Bunker CH (2011). Chromosome 8q24 variants are associated with prostate cancer risk in a high risk population of African ancestry. Prostate.

[67] Daraei A, Salehi R, Salehi M, Emami MH, Janghorbani M, Mohamadhashem F, Tavakoli H (2012). Effect of rs6983267 polymorphism in the 8q24 region and rs4444903 polymorphism in EGF gene on the risk of sporadic colorectal cancer in Iranian population. Med Oncol.

[68] Papanikolopoulou A, Landt O, Ntoumas K, Bolomitis S, Tyritzis SI, Constantinides C, Drakoulis N (2011). The multi-cancer marker, rs6983267, located at region 3 of chromosome 8q24, is associated with prostate cancer in Greek patients but does not contribute to the aggressiveness of the disease. Clin Chem Lab Med.

[69] Jones AM, Howarth KM, Martin L, Gorman M, Mihai R, Moss L, Auton A, Lemon C, Mehanna H, Mohan H, Clarke SE, Wadsley J, Macias E (2012). Thyroid cancer susceptibility polymorphisms: confirmation of loci on chromosomes 9q22 and 14q13, validation of a recessive 8q24 locus and failure to replicate a locus on 5q24. J Med Genet.

[70] Mates IN, Jinga V, Csiki IE, Mates D, Dinu D, Constantin A, Jinga M (2012). Single nucleotide polymorphisms in colorectal cancer: associations with tumor site and TNM stage. J Gastrointestin Liver Dis.

[71] Chan JY, Li H, Singh O, Mahajan A, Ramasamy S, Subramaniyan K, Kanesvaran R, Sim HG, Chong TW, Teo YY, Chia SE, Tan MH, Chowbay B (2013). 8q24 and 17q prostate cancer susceptibility loci in a multiethnic Asian cohort. Urol Oncol.

[72] Joung JY, Park S, Yoon H, Lee SJ, Park WS, Seo HK, Chung J, Kim SY, Hong SH, Lee YS, Kim J, Lee KH (2012). Association of common variations of 8q24 with the risk of prostate cancer in Koreans and a review of the Asian population. BJU Int.

[73] Ho CK, Halley L, Wei J, Habib FK (2012). Analysis of prostate cancer association with four single-nucleotide polymorphisms from genome-wide studies and serum phyto-estrogen concentrations. Prostate Cancer Prostatic Dis.

[74] Zhang X, Chen Q, He C, Mao W, Zhang L, Xu X, Zhu J, Chen B (2012). Polymorphisms on 8q24 are associated with lung cancer risk and survival in Han Chinese. PLoS One.

[75] Li FX, Yang XX, Hu NY, Du HY, Ma Q, Li M (2012). Single-nucleotide polymorphism associations for colorectal cancer in southern chinese population. Chin J Cancer Res.

[76] Win AK, Hopper JL, Buchanan DD, Young JP, Tenesa A, Dowty JG, Giles GG, Goldblatt J, Winship I, Boussioutas A, Young GP, Parry S, Baron JA (2013). Are the common genetic variants associated with colorectal cancer risk for DNA mismatch repair gene mutation carriers?. Eur J Cancer.

[77] Brankovic AS, Brajuskovic GN, Mircetic JD, Nikolic ZZ, Kalaba PB, Vukotic VD, Tomovic SM, Cerovic SJ, Radojicic ZA, Savic-Pavicevic DL, Romac SP (2013). Common variants at 8q24 are associated with prostate cancer risk in Serbian population. Pathol Oncol Res.

[78] Oskina NA, Boyarskikh UA, Lazarev AF, Petrova VD, Ganov DI, Tonacheva OG, Lifshits GI, Filipenko ML (2014). A replication study examining association of rs6983267, rs10090154, and rs1447295 common single nucleotide polymorphisms in 8q24 region with prostate cancer in Siberians. Urol Oncol.

[79] Yang B, Thyagarajan B, Gross MD, Goodman M, Sun YV, Bostick RM (2014). Genetic variants at chromosome 8q24, colorectal epithelial cell proliferation, and risk for incident, sporadic colorectal adenomas. Mol Carcinog.

[80] Cipollini M, Figlioli G, Garritano S, Bramante S, Maiorano L, Gnudi F, Cecchini A, De Paola F, Damicis L, Frixa T, Landi D, Cancemi L, De Santi C (2013). Risk of differentiated thyroid carcinoma and polymorphisms within the susceptibility cancer region 8q24. Cancer Epidemiol Biomarkers Prev.

[81] Yang B, Thyagarajan B, Gross MD, Fedirko V, Goodman M, Bostick RM (2014). No evidence that associations of incident, sporadic colorectal adenoma with its major modifiable risk factors differ by chromosome 8q24 region rs6983267 genotype. Mol Carcinog.

[82] San Francisco IF, Rojas PA, Torres-Estay V, Smalley S, Cerda-Infante J, Montecinos VP, Hurtado C, Godoy AS (2014). Association of RNASEL and 8q24 variants with the presence and aggressiveness of hereditary and sporadic prostate cancer in a Hispanic population. J Cell Mol Med.

[83] Nan H, Morikawa T, Suuriniemi M, Imamura Y, Werner L, Kuchiba A, Yamauchi M, Hunter DJ, Kraft P, Giovannucci EL, Fuchs CS, Ogino S, Freedman ML (2013). Aspirin use, 8q24 single nucleotide polymorphism rs6983267, and colorectal cancer according to CTNNB1 alterations. J Natl Cancer Inst.

[84] Haerian MS, Haerian BS, Rooki H, Molanaei S, Kosari F, Obohhat M, Hosseinpour P, Azimzadeh P, Mohebbi SR, Akbari Z, Zali MR (2014). Association of 8q24.21 rs10505477-rs6983267 haplotype and age at diagnosis of colorectal cancer. Asian Pac J Cancer Prev.

[85] Zhou CP, Pan HZ, Li FX, Hu NY, Li M, Yang XX (2014). Association analysis of colorectal cancer susceptibility variants with gastric cancer in a Chinese Han population. Genet Mol Res.

[86] Kurlapska A, Serrano-Fernandez P, Baszuk P, Gupta S, Starzynska T, Malecka-Panas E, Dabrowski A, Debniak T, Kurzawski G, Suchy J, Rogoza-Mateja W, Scott RJ, Lubinski J (2015). Cumulative effects of genetic markers and the detection of advanced colorectal neoplasias by population screening. Clin Genet.

[87] Labrador L, Torres K, Camargo M, Santiago L, Valderrama E, Chiurillo MA (2015). Association of common variants on chromosome 8q24 with gastric cancer in Venezuelan patients. Gene.

[88] Sahasrabudhe R, Estrada A, Lott P, Martin L, Polanco Echeverry G, Velez A, Neta G, Takahasi M, Saenko V, Mitsutake N, Jaeguer E, Duque CS, Rios A (2015). The 8q24 rs6983267G variant is associated with increased thyroid cancer risk. Endocr Relat Cancer.

[89] Zhao X, Wei X, Zhao L, Shi L, Cheng J, Kang S, Zhang H, Zhang J, Li L, Zhang H, Zhao W (2016). The rs6983267 SNP and long non-coding RNA CARLo-5 are associated with endometrial carcinoma. Environ Mol Mutagen.

[90] Risch N, Merikangas K (1996). The future of genetic studies of complex human diseases. Science.

[91] Li Q, Liu X, Hua RX, Wang F, An H, Zhang W, Zhu JH (2015). Association of three 8q24 polymorphisms with prostate cancer susceptibility: evidence from a meta-analysis with 50,854 subjects. Sci Rep.

[92] Zhang Y, Zeng X, Lu H, Ji H, Zhao E, Li Y (2016). Association between 8q24 (rs13281615 and rs6983267) polymorphism and breast cancer susceptibility: a meta-analysis involving 117,355 subjects. Oncotarget.

[93] Real LM, Ruiz A, Gayan J, Gonzalez-Perez A, Saez ME, Ramirez-Lorca R, Moron FJ, Velasco J, Marginet-Flinch R, Musulen E, Carrasco JM, Moreno-Rey C, Vazquez E (2014). A colorectal cancer susceptibility new variant at 4q26 in the Spanish population identified by genome-wide association analysis. PLoS One.

[94] Ioannidis JP, Ntzani EE, Trikalinos TA, Contopoulos-Ioannidis DG (2001). Replication validity of genetic association studies. Nat Genet.

[95] Wacholder S, Chanock S, Garcia-Closas M, El Ghormli L, Rothman N (2004). Assessing the probability that a positive report is false: an approach for molecular epidemiology studies. J Natl Cancer Inst.

[96] Xue WQ, He YQ, Zhu JH, Ma JQ, He J, Jia WH (2014). Association of BRCA2 N372H polymorphism with cancer susceptibility: a comprehensive review and meta-analysis. Sci Rep.

[97] Wang Y, Wu XS, He J, Ma T, Lei W, Shen ZY (2016). A novel TP53 variant (rs78378222 A > C) in the polyadenylation signal is associated with increased cancer susceptibility: evidence from a meta-analysis. Oncotarget.

[98] Egger M, Davey Smith G, Schneider M, Minder C (1997). Bias in meta-analysis detected by a simple, graphical test. Bmj.

[99] Thomas DC, Clayton DG (2004). Betting odds and genetic associations. J Natl Cancer Inst.

[100] Sankhwar M, Sankhwar SN, Bansal SK, Gupta G, Rajender S (2016). Polymorphisms in the XPC gene affect urinary bladder cancer risk: a case-control study, meta-analyses and trial sequential analyses. Sci Rep.

