# Supplementary material for: Association between 8q24 rs6983267 polymorphism and cancer susceptibility: a meta-analysis involving 170,737 subjects
Source: Oncotarget. 2017 Jul 4;8(34):57421–39. doi: 10.18632/oncotarget.18960 (PMC5593654; doi:10.18632/oncotarget.18960)
Supplement: Supplementary file 1 [file oncotarget-08-57421-s001.pdf]

Association between 8q24 rs6983267 polymorphism and cancer susceptibility: a meta-analysis involving 170,737 subjects

SUPPLEMENTARY MATERIALS

Supplementary Table 1: Characteristics of the 78 studies included in this meta-analysis for the association between rs6983267 and cancer risk. See Supplementary_Table_1

Supplementary Table 2: False-positive report probability values for all significant associations between the rs6983267 polymorphism and cancer risk. See Supplementary_Table_2

Supplementary Table 3: The meta-regression results of the association between the rs6983267 polymorphism and cancer risk. See Supplementary_Table_3

Supplementary Table 4: The detailed search strategies of the association between the rs6983267 polymorphism and cancer risk

Database	Search strategy	
Pubmed	#1 Polymorphism, genetic<br>#2 Polymorphism*<br>#3 SNP<br>#4 Single nucleotide polymorphism<br>#5 Variant<br>#6 Mutation<br>#7 #1 OR #2 OR #3 OR #4 OR #5 OR #6<br>#8 rs6983267<br>#9 Neoplasms<br>#10 Cancer<br>#11 Carcino*<br>#12 #9 OR #10 OR #11<br>#13 #7 AND #8 AND #12	((Polymorphism, genetic) OR Polymorphism* OR SNP OR (Single nucleotide polymorphism) OR Variant OR Mutation) AND rs6983267 AND (Neoplasms OR Cancer OR Carcino*)
Embase	#1 'neoplasm'/exp<br>#2 cancer<br>#3 tumor<br>#4 carcinoma<br>#5 carcinogenesis<br>#6 #1 OR #2 OR #3 OR #4 OR #5<br>#7 rs6983267<br>#8 'single nucleotide polymorphism'/exp<br>#9 SNP<br>#10 polymorphism<br>#11 variant<br>#12 mutation<br>#13 #8 OR #9 OR #10 OR #11 OR #12<br>#14 #6 AND #7 AND #13	('neoplasm'/exp OR cancer OR tumor OR carcinoma OR carcinogenesis) AND rs6983267 AND ('single nucleotide polymorphism'/exp OR SNP OR polymorphism OR variant OR mutation)

Supplementary Table 5: Score of quality assessment

Criteria	Score
**Ascertainment of cancer case**	
Histopathologic confirmation	2
by patient medical record	1
Not described	0
**Representativeness of case**	
Selected from population cancer registry	2
Selected from hospital	1
No method of selection described	0
**Representativeness of control**	
Population-based	2
Hospital-based	1
Not described	0
**Control selection**	
Controls matched with cases by age and sex	2
Controls matched with cases only by age or by sex	1
Not matched or not descried	0
**Genotyping examination**	
Genotyping done blindly and quality control	2
Only genotyping done blindly or quality control	1
Unblinded and without quality control	0
**Total sample size**	
> 400	2
200–400	1
≤ 200	0
